# Supplementary material for: Signaling cascades and the importance of moonlight in coral broadcast mass spawning
Source: eLife. 2015 Dec 15;4:e09991. doi: 10.7554/eLife.09991 (PMC4721961; doi:10.7554/eLife.09991)
Supplement: Supplementary file 2. — Gene enrichments (false discovery rate<0.1) across GO categories are shown. GOseq was used to test for enriched GO categories. DOI: http://dx.doi.org/10.7554/eLife.09991.013 [file elife-09991-supp2.zip › 09991_ Supplementary_file_2.pdf]

**Supplementary file 2.** Gene ontology enrichment analysis for *Acropora millepora* gene variability between new moon and full moon days. Gene enrichments (False Discovery Rate<0.1) across GO categories are shown. GOseq was used to test for enriched GO categories.

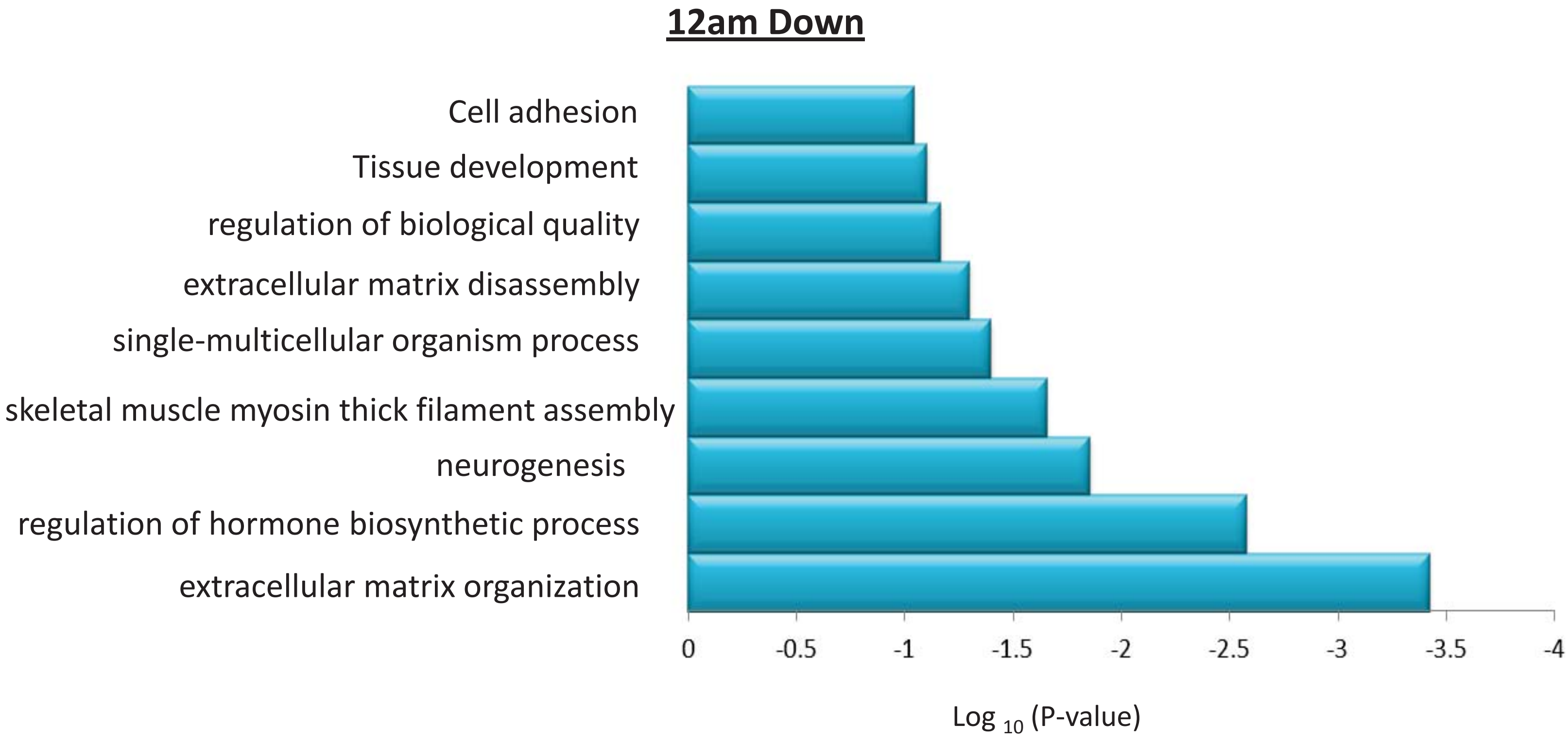

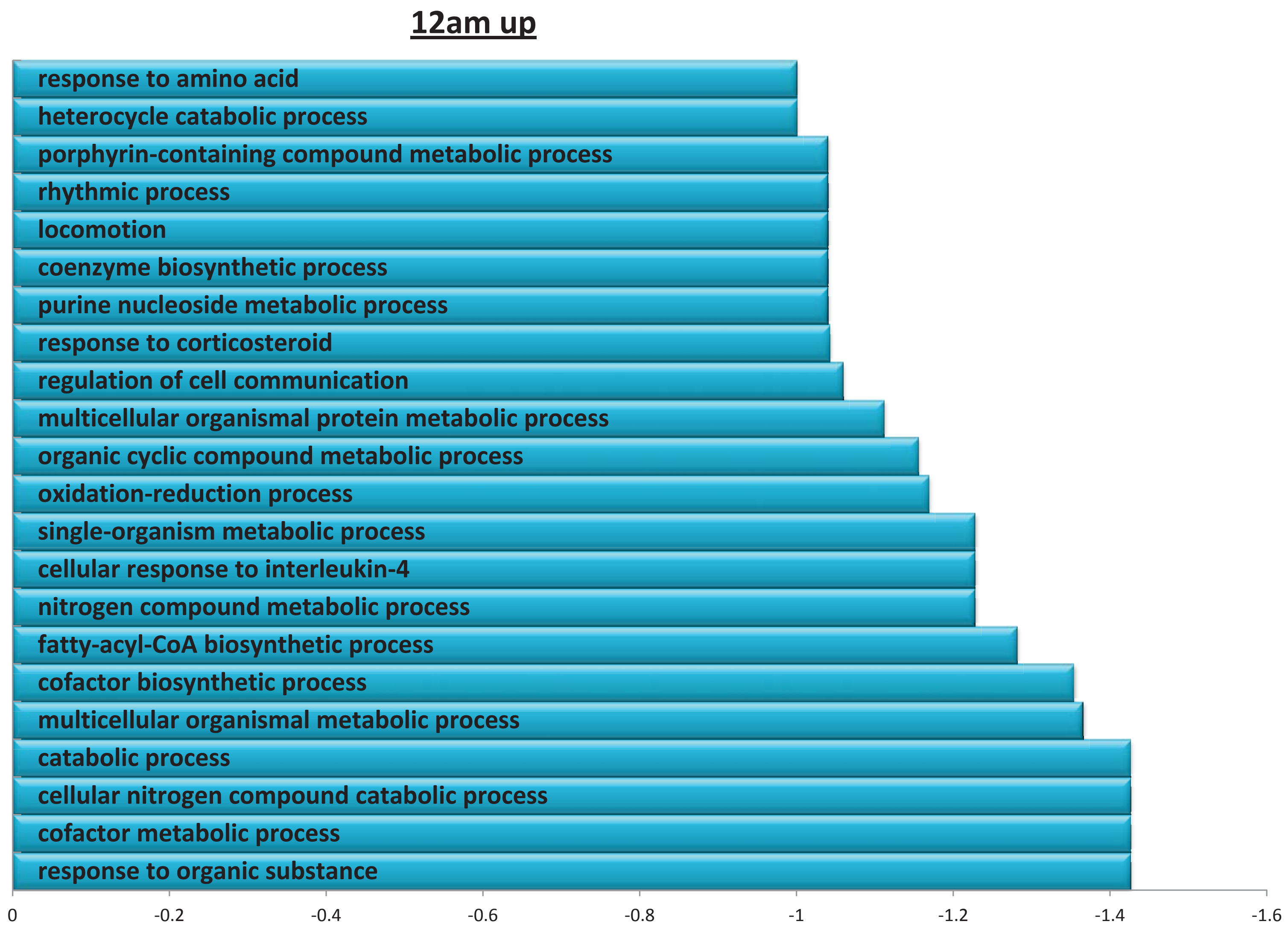
12am up
response to amino acid
heterocycle catabolic process
porphyrin-containing compound metabolic process
rhythmic process
locomotion
coenzyme biosynthetic process
purine nucleoside metabolic process
response to corticosteroid
regulation of cell communication
multicellular organismal protein metabolic process
organic cyclic compound metabolic process
oxidation-reduction process
single-organism metabolic process
cellular response to interleukin-4
nitrogen compound metabolic process
fatty-acyl-CoA biosynthetic process
cofactor biosynthetic process
multicellular organismal metabolic process
catabolic process
cellular nitrogen compound catabolic process
cofactor metabolic process
response to organic substance
0
-0.2
-0.4
-0.6
-0.8
-1
-1.2
-1.4
-1.6

## 6pm Down

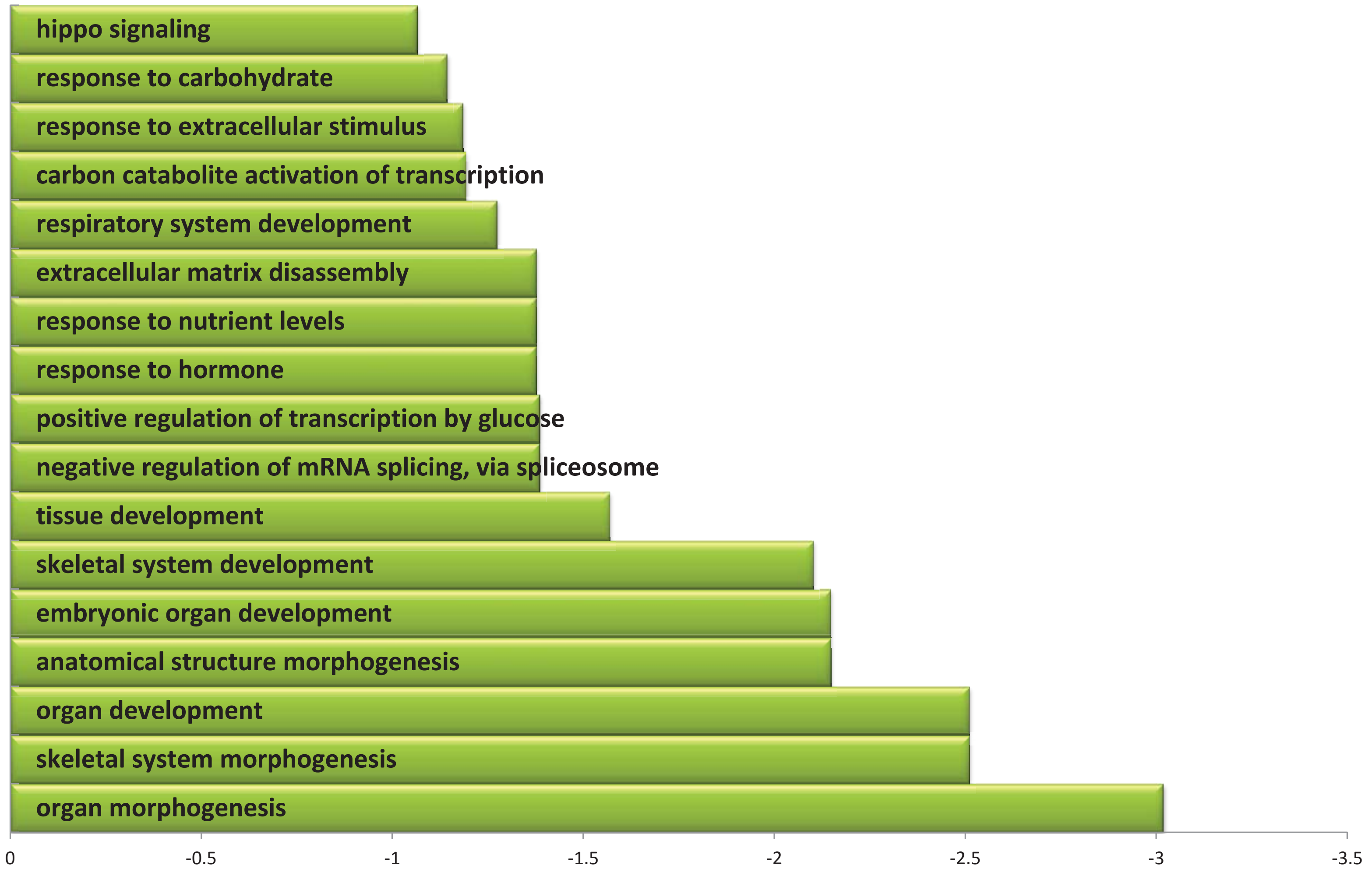

hippo signaling
response to carbohydrate
response to extracellular stimulus
carbon catabolite activation of transcription
respiratory system development
extracellular matrix disassembly
response to nutrient levels
response to hormone
positive regulation of transcription by glucose
negative regulation of mRNA splicing, via spliceosome
tissue development
skeletal system development
embryonic organ development
anatomical structure morphogenesis
organ development
skeletal system morphogenesis
organ morphogenesis
0
-0.5
-1
-1.5
-2
-2.5
-3
-3.5

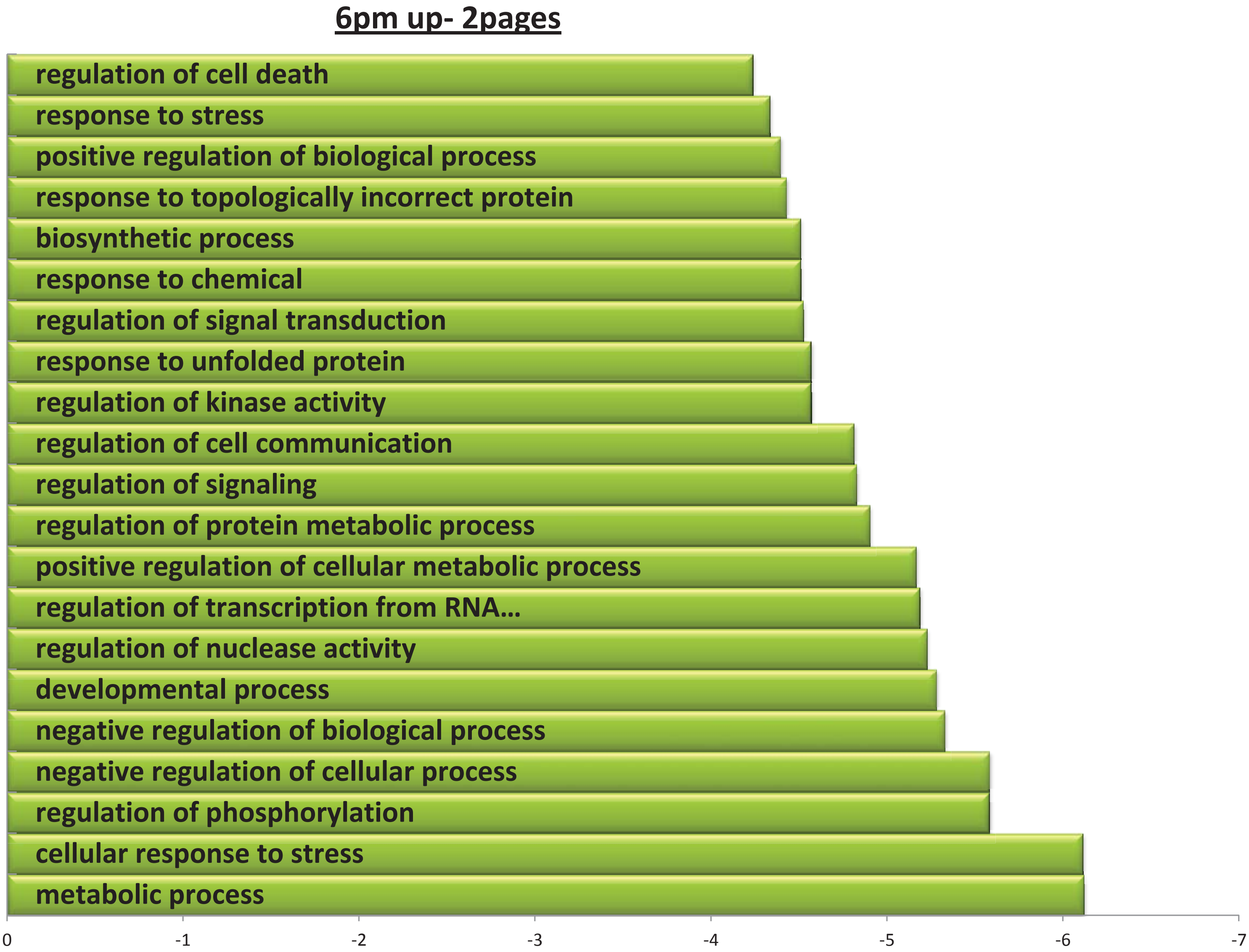
6pm up- 2pages
regulation of cell death
response to stress
positive regulation of biological process
response to topologically incorrect protein
biosynthetic process
response to chemical
regulation of signal transduction
response to unfolded protein
regulation of kinase activity
regulation of cell communication
regulation of signaling
regulation of protein metabolic process
positive regulation of cellular metabolic process
regulation of transcription from RNA...
regulation of nuclease activity
developmental process
negative regulation of biological process
negative regulation of cellular process
regulation of phosphorylation
cellular response to stress
metabolic process
0
-1
-2
-3
-4
-5
-6
-7

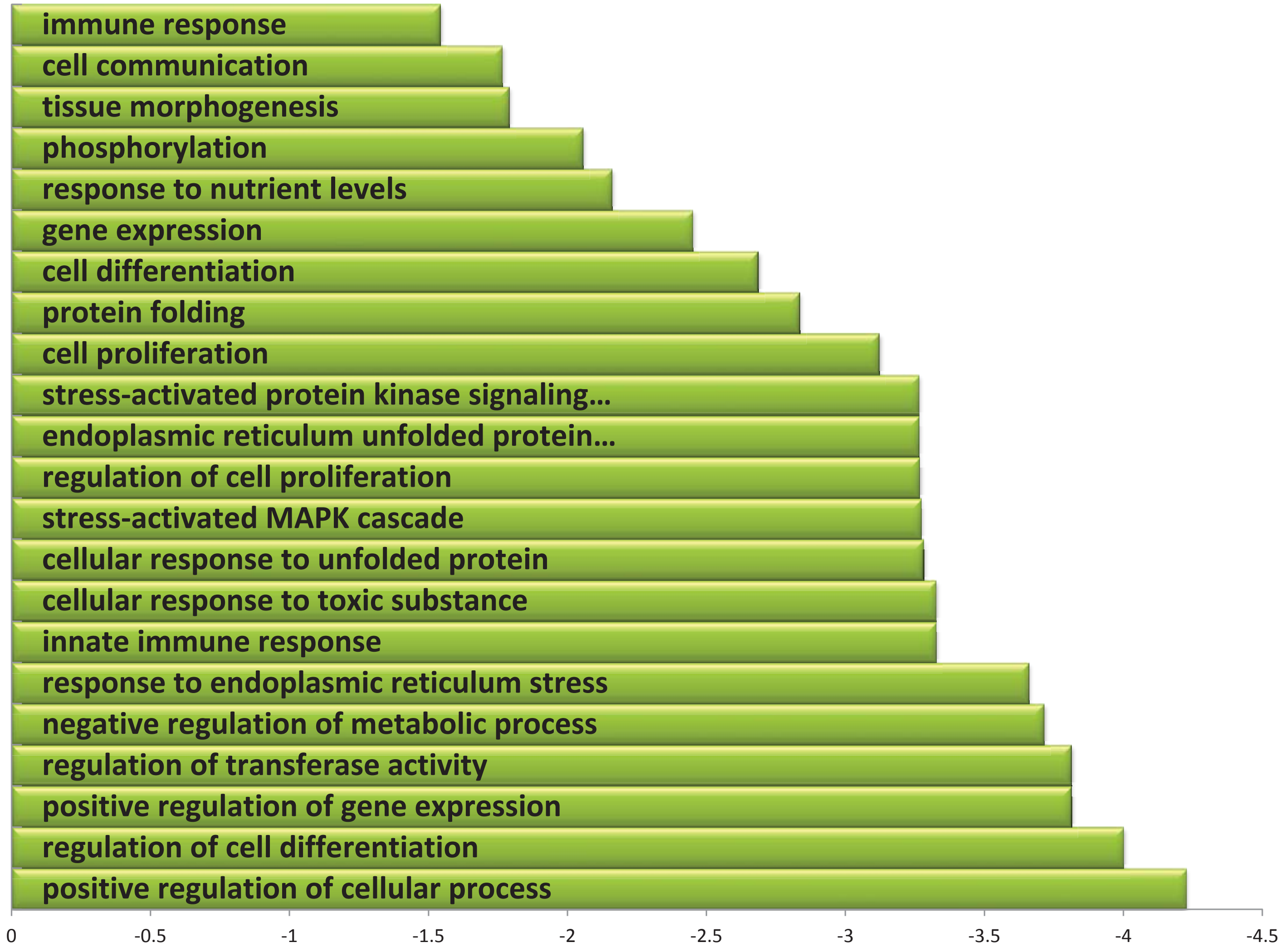

immune response
cell communication
tissue morphogenesis
phosphorylation
response to nutrient levels
gene expression
cell differentiation
protein folding
cell proliferation
stress-activated protein kinase signaling...
endoplasmic reticulum unfolded protein...
regulation of cell proliferation
stress-activated MAPK cascade
cellular response to unfolded protein
cellular response to toxic substance
innate immune response
response to endoplasmic reticulum stress
negative regulation of metabolic process
regulation of transferase activity
positive regulation of gene expression
regulation of cell differentiation
positive regulation of cellular process
0
-0.5
-1
-1.5
-2
-2.5
-3
-3.5
-4
-4.5

## 12pm down- 2 pages

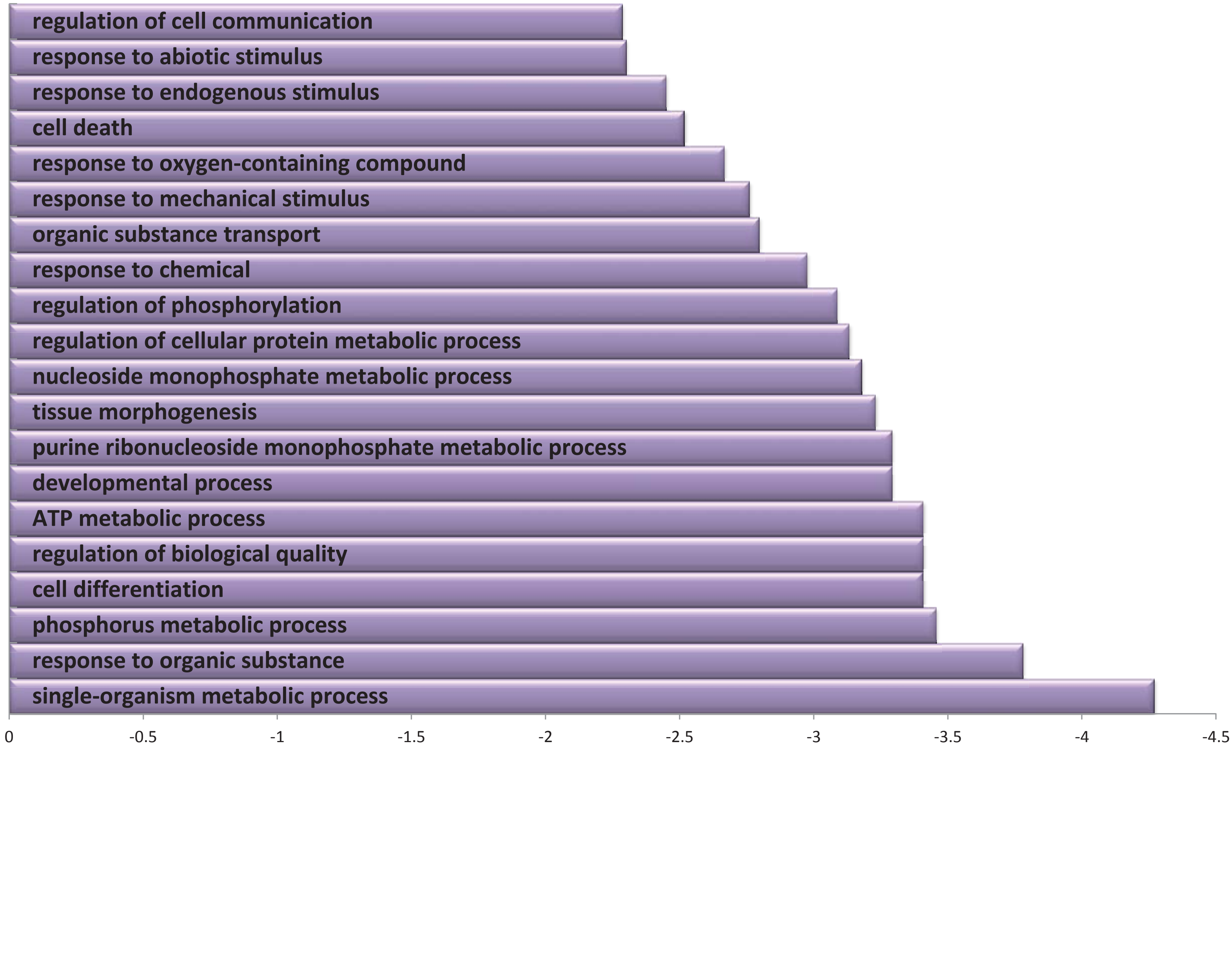

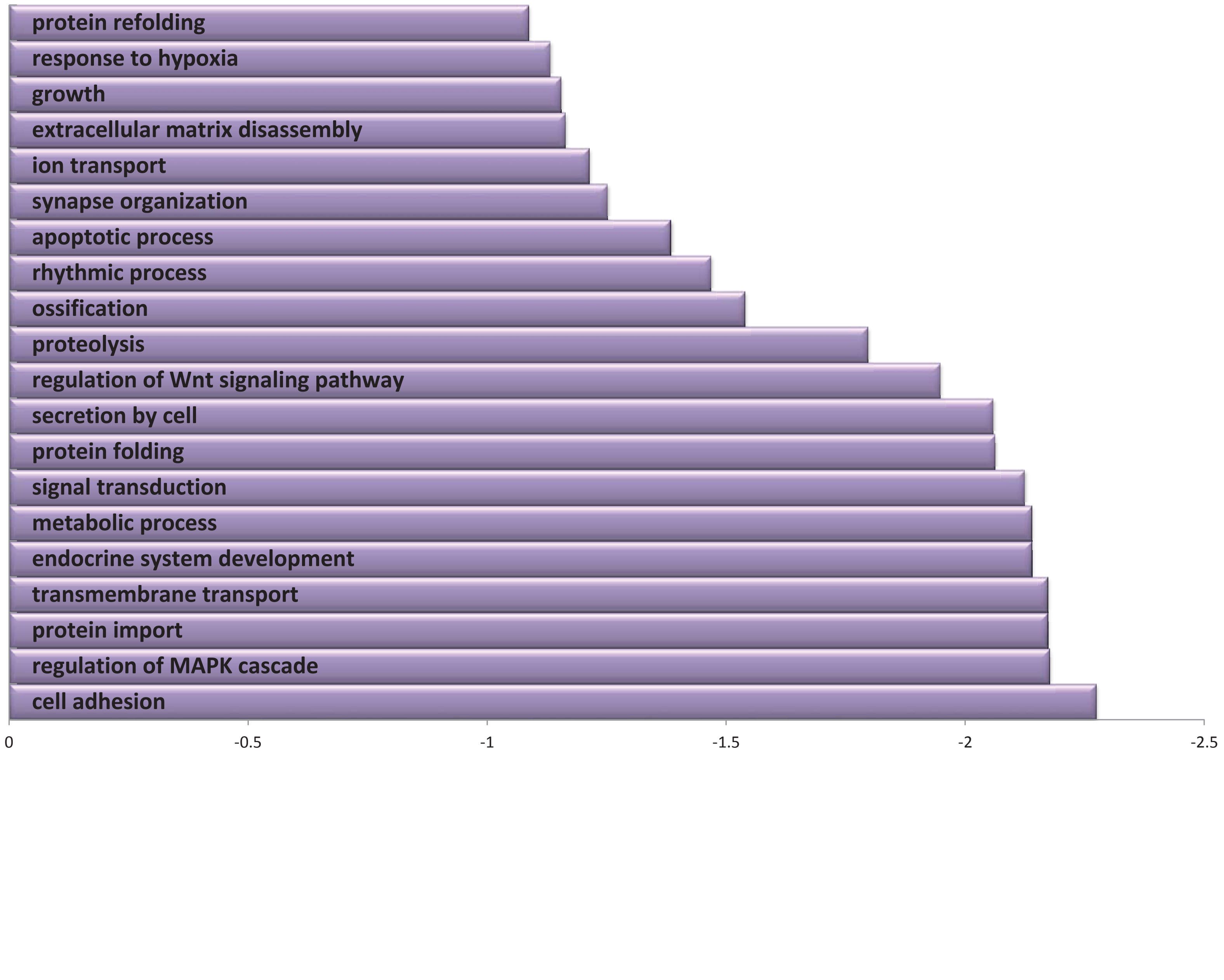
protein refolding
response to hypoxia
growth
extracellular matrix disassembly
ion transport
synapse organization
apoptotic process
rhythmic process
ossification
proteolysis
regulation of Wnt signaling pathway
secretion by cell
protein folding
signal transduction
metabolic process
endocrine system development
transmembrane transport
protein import
regulation of MAPK cascade
cell adhesion
0
-0.5
-1
-1.5
-2
-2.5

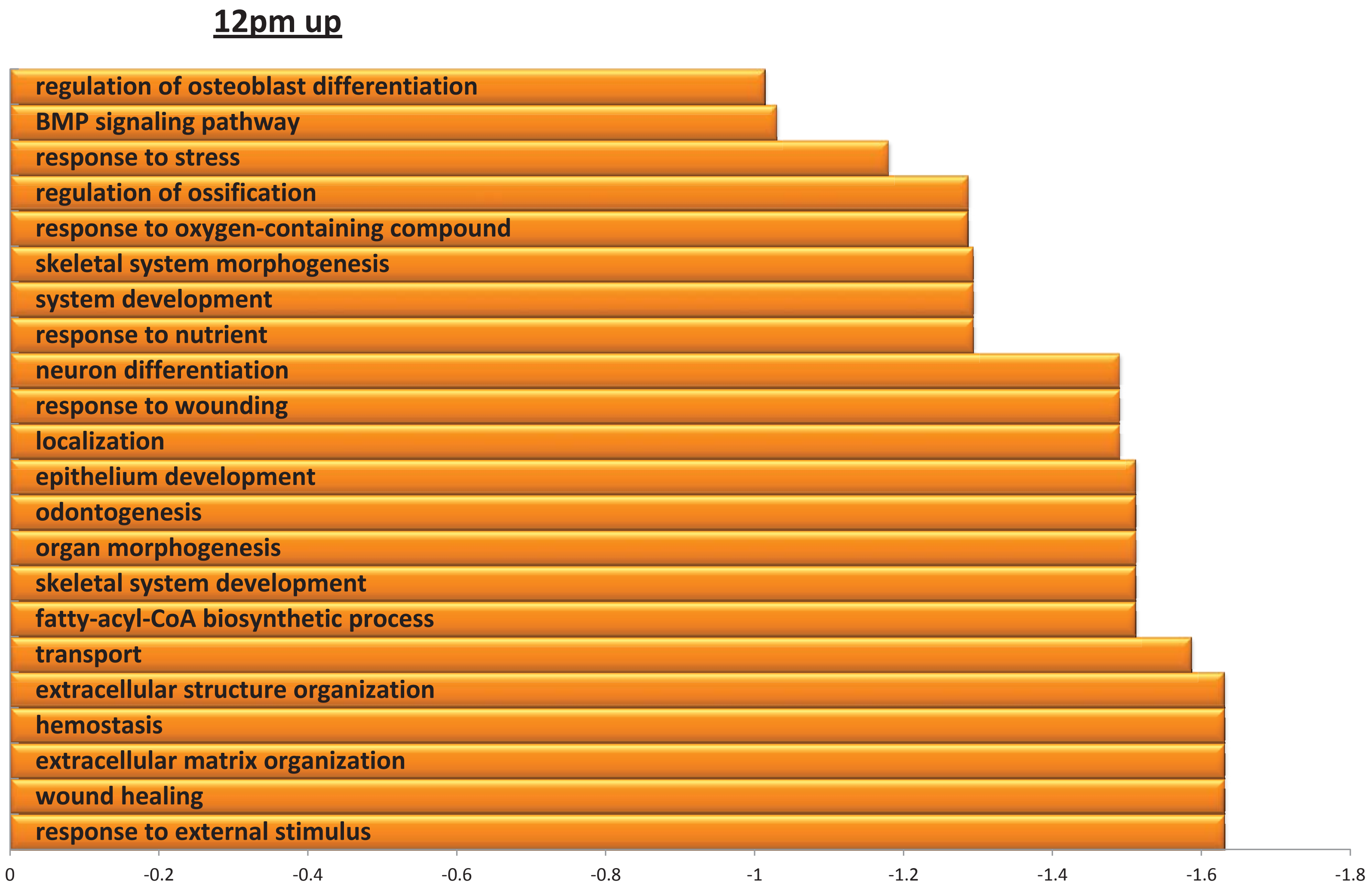
12pm up
regulation of osteoblast differentiation
BMP signaling pathway
response to stress
regulation of ossification
response to oxygen-containing compound
skeletal system morphogenesis
system development
response to nutrient
neuron differentiation
response to wounding
localization
epithelium development
odontogenesis
organ morphogenesis
skeletal system development
fatty-acyl-CoA biosynthetic process
transport
extracellular structure organization
hemostasis
extracellular matrix organization
wound healing
response to external stimulus
0
-0.2
-0.4
-0.6
-0.8
-1
-1.2
-1.4
-1.6
-1.8
