# Supplementary material for: Signaling cascades and the importance of moonlight in coral broadcast mass spawning
Source: eLife. 2015 Dec 15;4:e09991. doi: 10.7554/eLife.09991 (PMC4721961; doi:10.7554/eLife.09991)
Supplement: Supplementary file 3. — DOI: http://dx.doi.org/10.7554/eLife.09991.014 [file elife-09991-supp3.docx]

| Supplementary file 3. List of candidate genes used in qPCR expression analysis | | |  |
| --- | --- | --- | --- |
| **Name** | **Product size (bp)** | **Forward Primer 5'-3'** | **Reverse Primer 5'-3'** |
| Melanopsin B 1 | 100 | ACGTTGACAGAACGGCCAAA | TCTCCACAGGAGCGGAAATGA |
| Melanopsin B 2 | 100 | CTCCCCACCCTAATATAGGAGCAA | TACCCAGAAATCGTCACAACACAA |
| Neuropeptide Y receptor | 100 | AAGCAGAAAAGATCGCTACAGGAAA | CATCGGAATATTTGCAACCAATG |
| Pyroglutamylated RFamide peptide receptor | 104 | AGGACATTAAGTCCGTGCTTCCA | GATGAACGCCAACAAATCAAAAAT |
| Tachykinin-like peptides receptor | 100 | GGGACAGTTCAGGAACGGGATA | TTAGCTTTTCCGCTCGCTGTT |
| Synaptotagmin-7 | 100 | CTCCATGCTTCGCAAGAAAAG | GCGTTCAGAATGGGCTCAGA |
| Neuropeptide FF receptor | 100 | AACTTCAAACGGAAGCCTGCTT | TCTGTCCGTGTATCGGTTGAAGA |
| Enhancer of filamentation 1 | 103 | CTGACTCAGATTTACCTCAGAGCTTGA | AGAGGACGGTGTTGGAGTTCTTTG |
| Focal adhesion kinase 1 | 101 | GGCTCACAAAGTGTTGTCATCTGA | CGCGGCGGTATTCTTGGT |
| Melatonin receptor type 1B-B | 100 | TCCCTCATTGGCTGGACATG | GACTGCCTGAAAGAACGGTTGA |
| Cryptochrome-1 | 143 | GGCACGAGGATCATTGGAAAGGACTA | GTCTTTGCTTCCATCCGATTTCTCCG |
| Thyrotroph embryonic factor | 100 | CCGAAGTTCCCTCGTTTATTTGA | GCTGTACCAAAAGCAAGTGAATCCA |
| **Candidate reference genes** |  |  |  |
| Beta-actin | 200 | CTGATGGACAGGTCATCACCAT | CTCGTGGATACCAGCAGATTCC |
| Adeno-HomoCyase | 66 | CCTTGGATGTGCTATGGGTCA | GCCAAGACCTGGTTGGTGAA |
| Ribosomal protein L7 | 232 | GGAAAGGTCTCCAAACAGCGCACTGCC | CGCGGTTTCCATGATCACCGCCTTCC |
| Poly(a) binding protein | 167 | AATGGCGTCTCTATACGTTGG | CGTGAGCTGGTTGTTGGAA |
